# Wearable Sensor Assessment of Gait Characteristics in Individuals Awaiting Total Knee Arthroplasty: A Cross-Sectional, Observational Study

**DOI:** 10.3390/jfmk10030288

**Published:** 2025-07-28

**Authors:** Elina Gianzina, Christos K. Yiannakopoulos, Elias Armenis, Efstathios Chronopoulos

**Affiliations:** 1 School of Physical Education and Sport Science, National and Kapodistrian University of Athens, 17232 Athens, Greece; 2 Laboratory for Research of the Musculoskeletal System, “Th. Garofalidis”, KAT General Hospital, Medical School, National and Kapodistrian University of Athens, 14561 Athens, Greece

**Keywords:** gait impairments, knee osteoarthritis, total knee arthroplasty, wearable sensors, gait biomechanics

## Abstract

**Background:** Gait impairments are common in individuals with knee osteoarthritis awaiting total knee arthroplasty, affecting their mobility and quality of life. This study aimed to assess and compare biomechanical gait features between individuals awaiting total knee arthroplasty and healthy, non-arthritic controls, focusing on less-explored variables using sensor-based measurements. **Methods:** A cross-sectional observational study was conducted with 60 participants: 21 individuals awaiting total knee arthroplasty and 39 nonarthritic controls aged 64–85 years. Participants completed a standardized 14 m walk, and 17 biomechanical gait parameters were measured using the BTS G-Walk inertial sensor. Key variables, such as stride duration, cadence, symmetry indices, and pelvic angles, were analyzed for group differences. **Results:** The pre-total knee arthroplasty group exhibited significantly longer gait cycles and stride durations (*p* < 0.001), reduced cadence (*p* < 0.001), and lower gait cycle symmetry index (*p* < 0.001) than the control group. The pelvic angle symmetry indices for tilt (*p* = 0.014), rotation (*p* = 0.002), and obliquity (*p* < 0.001) were also lower. Additionally, the pre-total knee arthroplasty group had lower propulsion indices for both legs (*p* < 0.001) and a lower walking quality index on the right leg (*p* = 0.005). The number of elaborated steps was significantly greater in the pre-total knee arthroplasty group (left, *p* < 0.001, right: *p* < 0.001). No significant differences were observed in any other gait parameters. **Conclusions:** This study revealed significant gait impairment in individuals awaiting total knee arthroplasty. Although direct evidence for prehabilitation is lacking, future research should explore whether targeted approaches, such as strengthening exercises or gait retraining, can improve gait and functional outcomes before surgery.

## 1. Introduction

Knee osteoarthritis is a common chronic degenerative joint disease characterized by stiffness, discomfort, and reduced joint function. In its advanced stages, knee osteoarthritis leads to significant physical impairment, pain, and disability, severely restricting daily activities such as walking, standing, and climbing stairs [1,2,3]. Consequently, the burden of knee osteoarthritis is not only felt physically but also socially and psychologically, affecting overall well-being and productivity [4]

Total knee arthroplasty (TKA) is regarded as the most effective treatment for individuals with advanced knee osteoarthritis, as it alleviates symptoms and improves mobility [5,6,7]. However, patients awaiting TKA often experience substantial gait impairment, which compromises their mobility, balance, and stability [8,9]. These gait disturbances include reduced walking speed, prolonged gait cycle duration, and increased asymmetry, all of which contribute to an elevated risk of falls and decreased independence in performing activities of daily living [10,11,12].

Recent systematic reviews have emphasized the critical role of gait impairment as an indicator of functional decline in patients with knee osteoarthritis. For example, Favre and Jolles [13] provided a reference dataset on lower limb biomechanics in knee osteoarthritis, underscoring the importance of gait analysis in understanding the disease progression. Meta-analyses have also shown that reduced walking speed and asymmetry correlate with knee osteoarthritis severity and can predict outcomes after TKA [14].

Wearable sensor technology has emerged as a valuable tool for assessing and quantifying gait biomechanics in clinical and research settings. Devices such as the BTS G-Walk inertial sensor provide noninvasive, real-time evaluations of gait parameters, including cadence, gait cycle duration, stride length, and symmetry [15,16,17,18,19]. These sensors allow for detailed gait analysis, enabling clinicians to track gait abnormalities over time and develop targeted prehabilitation and rehabilitation strategies to improve functional outcomes before surgery [20,21].

The primary aim of this study was to evaluate the biomechanical gait features of pre-TKA individuals and compare them with those of healthy, nonarthritic controls using inertial sensor-based measurements. While previous studies have explored general gait impairments in patients with knee osteoarthritis, they have primarily concentrated on basic parameters such as gait speed and cadence [10,11,14,21,22]. This study further investigated less-studied parameters, particularly pelvic motion asymmetries (e.g., pelvic tilt, rotation, and obliquity) and the propulsion index, which provide new insights into the compensatory strategies employed by individuals before TKA. By using advanced wearable technology to track these parameters, we aimed to uncover compensatory movement patterns and biomechanical dysfunctions in the pre-TKA group before surgery. These findings may inform the development of targeted presurgical interventions to optimize postoperative recovery.

## 2. Materials and Methods

### 2.1. Participants

The study included 21 patients with primary knee osteoarthritis (3 males and 18 females) with a mean age of 73.57 ± 6.44 years and 39 volunteers without arthritis (10 males and 29 females) with a mean age of 71.59 ± 4.81 years. Cases and controls were matched individually based on age, sex, and body mass index (BMI), with each knee osteoarthritis patient paired with approximately 1.9 non-arthritic controls. Matching criteria were selected to control for potential confounding factors, such as age, sex, and BMI, which are known to affect gait performance and may influence study outcomes. Patients with end-stage unilateral primary knee osteoarthritis scheduled for TKA were recruited. The inclusion criteria required participants to walk independently without the use of ambulatory aids. Individuals with neurological, cardiorespiratory, or severe orthopedic conditions that impaired mobility were excluded. The normal non-arthritic group comprised healthy individuals with no history of orthopedic or neurological disorders, recent injuries, surgeries, or medications that could affect their gait or balance.

Before participation, all participants provided written informed consent after receiving a detailed explanation of the study objectives. This study adhered to the principles of the Declaration of Helsinki and was approved by the Institutional Review Board (IRB) of the School of Physical Education and Sport Science at the National and Kapodistrian University of Athens, Greece (Approval Number: 1306/22-09-2021).

### 2.2. Instrumentations

Gait analysis was conducted using the G-Walk wireless inertial sensor (BTS Bioengineering S.p.A., Milan, Italy), which integrates a triaxial accelerometer, gyroscope, and magnetometer. The G-Walk system has demonstrated high test–retest reliability (ICCs > 0.90) and strong agreement with motion capture systems for parameters such as gait cycle duration, cadence, and step length [15,16,17,18,19]. The algorithms used primarily rely on accelerometer and gyroscope data, with the magnetometer contributing to orientation tracking, particularly for pelvic rotation. All trials were performed on flat, nonmetallic indoor surfaces to minimize magnetic interference.

The triaxial accelerometer offers multiple sensitivity settings with dynamic ranges of ±2, ±4, ±8, and ±16 g, operating within a bandwidth of 4–1000 Hz and providing 16-bit precision per axis. The magnetometer features a 13-bit resolution, dynamic range of ±1200 μT, and bandwidth of up to 100 Hz. The gyroscope also provides 16-bit precision per axis, with sensitivity options of ±250, ±500, ±1000, and ±2000°/s and a bandwidth range of 4–8000 Hz.

Additionally, the module is equipped with a GPS receiver that ensures high positional accuracy, offering 2.5 m accuracy at up to 5 Hz or 3 m accuracy at up to 10 Hz with a bandwidth of up to 10 Hz.

In terms of dimensions, the device measures 70 mm × 40 mm × 18 mm (2.75 × 1.57 × 0.7 in) and supports acquisition frequencies of up to 1000 Hz. For data collection, it was connected to a laptop via Bluetooth 3.0, with a transmission range of up to 60 m in a direct line of sight.

### 2.3. Data Collection Protocol

During testing, the sensor was secured inside a semi-elastic black belt positioned over the participants’ S1–S2 vertebrae. This study was conducted in three sequential phases: (1) data collection, (2) analysis, and (3) interpretation of the results. These steps were completed without strict time tracking.

During the data collection phase, the participants followed a 14 m walking protocol. Prior to the test, they received clear instructions to ensure the consistency and reliability of the results. The test began with the participants standing still for a moment to allow for stabilization. Upon receiving a signal, the participants walked along a straight path at a steady and moderate pace. The objective was to complete at least five full gait cycles over a 14 m distance before turning around.

Before each turn, the participants paused for at least one second to regain stability and then turned and paused again for another second before proceeding in the opposite direction. This approach helps minimize variability while preserving natural walking patterns.

Each participant completed at least two trials of the 14 m walk, with additional trials permitted to ensure reliable data and confirm gait pattern consistency. As this was a cross-sectional study, no follow-up was required, and all data were collected at a single time point.

### 2.4. Data Analysis

Gait data were collected using Bluetooth 3.0 and transmitted in real time to a computer for further processing. G-Studio software (version 1.3.0) was used to analyze the data, applying automated filters to eliminate noise and correct inconsistencies in the walking patterns. The software then uses built-in algorithms to calculate the various spatiotemporal gait parameters. These algorithms transform raw motion sensor signals into clinically relevant gait metrics. Their accuracy was confirmed through internal calibration procedures and validation studies against gold-standard motion capture systems. Previous studies have also shown strong correlations between parameters calculated from wearable sensors and directly measured joint kinematics, supporting the reliability of this approach [15,16,17,18,19].

The extracted gait parameters were categorized into three main groups:

1. Global analysis parameters

These parameters provide an overview of the walking pattern and include the following:

- Cadence (steps/min): number of steps taken per minute.
- Speed (m/s): average walking velocity.
- Symmetry index of the gait cycle: this index measures the percentage of symmetry between the anterior and posterior acceleration curves during the right and left gait cycles.
- Symmetry index of pelvic angles (tilt, obliquity, rotation): This index assesses the percentage similarity or difference in pelvic movements recorded during the right and left gait cycles. These angles were measured in three anatomical planes—sagittal (tilt), frontal (obliquity), and transverse (rotation) planes.

2. Side-specific parameters (Left and Right)

These parameters focus on the individual foot movements and gait cycle dynamics for each leg:

- Stride length (m): the average distance covered between consecutive initial contacts of the same foot.
- Stride length as a percentage of height (% height): normalized stride length relative to the participant’s height.
- Gait cycle duration (s): time interval between two consecutive heel strikes of the same foot.
- Step length (% stride length): the average distance between the initial foot contact and the next contact made by the opposite foot.
- Stance phase (% cycle): percentage of the gait cycle during which the foot remains in contact with the ground.
- Swing phase (% cycle): the percentage of the gait cycle during which the foot is in motion and not in contact with the ground.
- Double support phase (% cycle): percentage of the gait cycle in which both feet are simultaneously in contact with the ground.
- Single support phase (% cycle): percentage of the gait cycle in which only one foot is in contact with the ground.
- Elaborated steps: total number of strides considered in the analysis.
- Propulsion index: represents the inclination of the line following the rising edge of the acceleration pattern.

3. Walk quality index

The walk quality index is a composite measure that evaluates gait efficiency, stability, and symmetry by integrating key gait parameters such as step length, cadence, symmetry, and variability. A higher score indicates a more efficient, stable, and symmetrical walking pattern, whereas a lower score indicates a less efficient, unstable, or more variable gait.

### 2.5. Statistical Analysis

All statistical analyses were performed using IBM SPSS Statistics version 29.0 (IBM Corporation, Somers, NY, USA). Data are presented as mean ± standard deviation (SD) or, for non-normally distributed variables, as the median with interquartile range (IQR). The Kolmogorov–Smirnov and Shapiro–Wilk tests were used to assess the normality of the data.

To compare the groups, an independent samples *t*-test and Fisher’s exact test were used to evaluate homogeneity between the groups. If the normality assumptions were violated, the Mann–Whitney U test was used to analyze the differences between the groups. Effect sizes were reported alongside *p*-values: Cohen’s d for *t*-tests and rank-biserial correlation (r) for Mann–Whitney U tests, providing a more comprehensive understanding of the practical significance of the observed differences.

A priori power analysis was conducted using GPower (version 3.1) to determine the required sample size for detecting large effect sizes (Cohen’s d ≈ 0.8) at a significance level of *p* < 0.05, with a target power of 80%. All statistical tests were two-tailed, with significance set at *p* < 0.05.

## 3. Results

The demographic characteristics of the participants are presented in Table 1. The pre-TKA group had a mean age of 73.57 ± 6.44 years, whereas the normal non-arthritic group had a mean age of 71.59 ± 4.81 years, with no significant difference between the groups (*p* = 0.229). Similarly, no significant differences were observed in weight (pre-TKA: 82.29 ± 16.92 kg, normal: 77.96 ± 9.66 kg; *p* = 0.270), height (pre-TKA: 165.52 ± 7.23 cm, normal: 163.81 ± 7.45 cm; *p* = 0.429), BMI (pre-TKA: 30.01 ± 5.75, normal: 29.15 ± 3.97; *p* = 0.541), or shoe size (pre-TKA: 39.76 ± 2.26, normal: 39.81 ± 3.98; *p* = 0.332). Sex distribution was comparable among the groups (*p* = 0.704).

Table 2 summarizes the gait parameters of the two groups. Significant differences were observed in several gait parameters. First, the gait cycle duration was significantly longer in the pre-TKA group than in the normal group for both the left (1.36 ± 0.14 vs. 1.11 ± 0.06 s, *p* < 0.001, Cohen’s d = 2.17) and right (1.35 ± 0.14 vs. 1.11 ± 0.07 s, *p* < 0.001, Cohen’s d = 2.17) sides, with large effect sizes, indicating substantial differences. Similarly, stride duration was significantly longer in the pre-TKA group for both the left (1.37 ± 0.16 vs. 1.11 ± 0.06 s, *p*< 0.001, Cohen’s d = 2.15) and right (1.36 ± 0.16 vs. 1.11 ± 0.07 s, *p* < 0.001, Cohen’s d = 2.02) legs.

In addition, cadence was significantly lower in the pre-TKA group (91.12 ± 9.02 vs. 108.78 ± 6.64 steps/min, *p* < 0.001, Cohen’s d = −2.23) with a large effect size. Similarly, walking speed was significantly slower in the pre-TKA group (0.87 ± 0.16 vs. 1.11 ± 0.14 m/s, *p* < 0.001, Cohen’s d = −1.60).

However, no significant differences were observed in stance duration for the left (60.95 ± 5.16 vs. 60.80 ± 2.34%, *p* = 0.892, Cohen’s d = 0.04) or right (60.66 ± 5.07 vs. 60.15 ± 2.90%, *p* = 0.663, Cohen’s d = 0.12) side, with very small effect sizes. Likewise, no significant differences were found in stride length for the left (1.18 ± 0.20 vs. 1.23 ± 0.15 m, *p* = 0.274, Cohen’s d = −0.28) or right (1.18 ± 0.19 vs. 1.23 ± 0.15 m, *p* = 0.277, Cohen’s d = −0.29) sides.

Moreover, the first left double support was not significantly different between the groups (11.18 ± 2.02 vs. 10.26 ± 2.38%, *p* = 0.163, Cohen’s d = 0.42). Similarly, no significant differences were found in the left single support (39.14 ± 4.88 vs. 39.65 ± 2.67%, *p* = 0.646, Cohen’s d = −0.13) or right single support (38.79 ± 5.22 vs. 39.10 ± 0.91%, *p* = 0.773, Cohen’s d = −0.08) times. The right swing duration was not significantly different between the groups (39.34± 5.07 vs. 39.85 ± 2.90%, *p* = 0.663, Cohen’s d = −0.12).

In contrast, the left (3.78 ± 1.47 vs. 6.45 ± 1.30, *p* < 0.001, Cohen’s d = −1.92) and right (4.01 ± 1.58 vs. 6.45 ± 1.42, *p* < 0.001, Cohen’s d = −1.62) propulsion indices were significantly lower in the pre-TKA group, with large effect sizes. Furthermore, the right walk quality index was significantly lower in the pre-TKA group (91.47 ± 5.30 vs. 94.91 ± 2.65, *p* = 0.005, Cohen’s d = −0.82).

The elaborated steps were significantly longer in the pre-TKA group for both the left (11.00 ± 6.00 vs. 8.00 ± 2.00 s, *p* < 0.001, r = 0.66) and right (11.00 ± 5.00 vs. 8.00 ± 2.00 s, *p* < 0.001, r = 0.69) sides, with moderate effect sizes. However, the symmetry index of the gait cycle was significantly lower in the pre-TKA group (92.7 ± 14.85 vs. 97.0 ± 2.4, *p* < 0.001, r = 0.71), indicating more asymmetrical gait patterns in the pre-TKA group.

Regarding stride length as a percentage of height, there were no significant differences between the left (69.1 ± 18.25 vs. 75.9 ± 11.7%, *p* = 0.143, r = 0.17) and right (69.5 ± 17.65 vs. 75.4 ± 10.9%, *p* = 0.132, r = 0.18). Similarly, the left swing duration showed no significant difference (39.3 ± 6.6 vs. 38.5 ± 3.2%, *p* = 0.893, r = 0.01). The first right double support was not significantly different (10.2 ± 3.1 vs. 11.8 ± 4.3%, *p* = 0.417, r = 0.08).

Finally, the left walk quality index was significantly lower in the pre-TKA group (93.5 ± 6.8 vs. 95.9 ± 4.3, *p* = 0.036, r = 0.21). In addition, significant differences were observed in the symmetry indices of pelvic angles for tilt (50.5 ± 49.1 vs. 74.2 ± 27.4, *p* = 0.014, r = 0.28), obliquity (90.3 ± 9.8 vs. 98.7 ± 0.8, *p* < 0.001, r = 0.52), and rotation (94.8 ± 4.1 vs. 98.1 ± 2.4, *p* = 0.002, r = 0.40), with moderate-to-large effect sizes, indicating more asymmetrical gait patterns in the pre-TKA group.

## 4. Discussion

This study compared the gait characteristics between individuals pre-TKA and those in a normal non-arthritic group, providing valuable baseline data for future research. The findings revealed significant differences in gait cycle duration, cadence, stride duration, propulsion indices, symmetry, and other gait phase alterations, with large effect sizes for many variables, indicating substantial gait impairment in the pre-TKA group. These results underscore the significant gait impairments in pre-TKA individuals, which could affect their mobility and functional performance [23].

In particular, the pre-TKA group exhibited significantly longer gait cycle and stride durations (*p* < 0.001), indicating a slower walking pattern, which is consistent with previous findings in patients with knee osteoarthritis [24,25]. These results support the view that individuals with knee osteoarthritis often adopt cautious and pain-avoiding gait strategies. However, stride length did not differ significantly between the groups (left, *p* = 0.274; right, *p* = 0.277), implying that the slower pace is primarily driven by reduced cadence rather than altered step length [26,27].

Indeed, cadence was significantly lower in the pre-TKA group (91.12 ± 9.02 vs. 108.78 ± 6.64 steps/min, *p* < 0.001, Cohen’s d = −2.23), reinforcing the trend toward a more cautious walking pattern to minimize joint strain [28,29]. Gait speed was also significantly lower (0.87 ± 0.16 m/s vs. 1.11 ± 0.14 m/s, Cohen’s d = −1.60), reflecting further functional decline before surgery. These observations point to meaningful mobility limitations in this population, although the causal benefits of prehabilitation remain to be established.

Pelvic angle symmetry was significantly reduced in the pre-TKA group, with lower symmetry indices for tilt (*p* = 0.014, d = −0.28), obliquity (*p* < 0.001, d = −0.52), and rotation (*p* = 0.002, d = −0.40). These asymmetries likely reflect compensatory adaptations to offloading the affected limbs. Such strategies may lead to muscular imbalance and joint overloading, contributing to postoperative dysfunction [30,31]. Gait cycle symmetry was also significantly compromised (92.7 ± 14.85 vs. 97.0 ± 2.4, *p* = 0.000, r = 0.71), indicating widespread gait irregularities that could predispose patients to further musculoskeletal problems.

Additionally, the significantly lower propulsion index in the pre-TKA group for both the left (Cohen’s d = −1.92) and right (Cohen’s d = −1.62) limbs further support the idea that individuals with knee osteoarthritis have compromised gait mechanics. Reduced propulsion reflects diminished force generation during the push-off phase, indicating impaired walking dynamics [32,33]. The walk quality index also showed significant reductions on the right side (*p* = 0.005, Cohen’s d = −0.82) and a marginal reduction on the left side (*p* = 0.036, Cohen’s d = −0.21), further emphasizing the impaired overall gait quality in the pre-TKA group.

Notably, the elaborated steps (left, *p* = 0.000, Cohen’s r = 0.66; right, *p* = 0.000, Cohen’s r = 0.69) were significantly longer in the pre-TKA group, suggesting altered stepping strategies possibly linked to pain or compensation for mobility impairment.

No significant differences were observed in stance duration, single support phase, or stride length between the pre-TKA and control groups. These nonsignificant findings suggest that gait impairments in the pre-TKA group may be primarily due to reduced cadence rather than changes in step length or gait phase duration.

The findings of reduced cadence, propulsion, and pelvic symmetry in this study are consistent with previous evidence that individuals with knee osteoarthritis adopt compensatory gait patterns to mitigate pain and maintain stability [22,26]. These maladaptive patterns can lead to muscle imbalances, reduced mobility, and elevated fall risk, underscoring the importance of early targeted intervention. Evidence supports the effectiveness of strengthening exercises, particularly of the quadriceps and hip abductors, in improving gait biomechanics in this population [34]. In addition, wearable sensor-based biofeedback, such as real-time gait monitoring using inertial measurement units, may help to personalize rehabilitation and improve motor learning [20,21].

Although the current study did not assess the impact of prehabilitation, the observed biomechanical deficits provide a strong rationale for future studies. Recommended strategies include pelvic stabilization and trunk control exercises to address asymmetries in tilt, obliquity, and rotation; strengthening of the hip musculature to enhance stance phase stability; and eccentric quadriceps training to support deceleration and propulsion during gait transitions [35]. Real-time biofeedback can further enhance outcomes by providing visual or auditory cues to correct asymmetry and improve temporal parameters. Collectively, these findings support the prioritization of individualized prehabilitation programs to optimize mobility in patients awaiting TKA.

## 5. Limitations

Although the findings of this study provide valuable insights into gait mechanics in individuals with severe knee osteoarthritis awaiting TKA, several limitations should be acknowledged.

The pre-TKA and non-arthritic control groups comprised 21 and 39 participants, respectively. Although the sample sizes were adequate for detecting large effect sizes (Cohen’s d ≈ 0.8) with 80% power, the unequal sample sizes between the groups may limit the statistical robustness of certain comparisons, especially for subgroup analyses (e.g., those based on sex). Future research should aim for larger and more balanced sample sizes to enhance statistical power, improve generalizability, and reduce potential bias.

The cross-sectional design of this study restricts the ability to infer causal relationships and observe longitudinal changes over time. Although this study provides a snapshot of gait characteristics in a specific population, it does not allow us to assess how gait patterns evolve with the progression of knee osteoarthritis, after surgery, or in response to rehabilitation intervention. Longitudinal studies are needed to understand how gait mechanics change over time and in response to clinical treatments.

The study sample consisted of patients with knee osteoarthritis awaiting total knee arthroplasty and a nonarthritic control group, which may not fully represent the general population. The findings may be specific to individuals with knee osteoarthritis awaiting surgery and may not apply to other populations with different health conditions, ethnic backgrounds, or lifestyle factors. Future studies should consider diverse groups to increase the generalizability of their findings.

Although the BTS G-Walk sensor system is a reliable tool for gait analysis, its placement on the lower back may not fully capture lower limb kinematics, particularly joint-specific data. This limitation affects the comprehensiveness of the gait analysis. Future studies using multisensor systems, including sensors on the foot or knee, could provide a more detailed and accurate understanding of gait mechanics and asymmetries.

Additionally, despite the BTS G-Walk sensor system offering a convenient and reliable method for assessing gait, pelvic rotation measurements may be influenced by magnetic disturbances in indoor environments, especially when metallic objects are present. Although data were collected on a clear, non-metallic indoor surface to minimize this risk, some variability in the rotation symmetry measures may persist. Future studies should consider applying magnetometer calibration procedures or comparing alternative sensor placements to enhance accuracy.

While the use of Welch’s *t*-test helped correct for violations of the assumption of equal variances, some assumptions of the *t*-tests (such as normality) may still have influenced the effect size estimates and statistical conclusions. Although the Kolmogorov–Smirnov and Shapiro–Wilk tests were used to assess the normality of the data, some deviations from normality could have remained, which may affect the reliability of the results. Future studies should consider employing more advanced statistical methods, such as mixed-model analyses, which can better account for variations and improve the robustness of the findings, particularly in cases where violations of the assumptions persist.

Addressing these limitations in future studies will improve the understanding of gait mechanics in individuals with knee OA and help refine clinical applications for rehabilitation, performance optimization, and postsurgical recovery.

## 6. Conclusions

This study highlighted significant gait impairments in individuals with knee osteoarthritis awaiting TKA, including slower walking speed, reduced cadence, and altered symmetry, compared with those in healthy controls. These findings suggest that individuals in the pre-TKA group adopt compensatory gait strategies that potentially affect their mobility and functional performance negatively. Although this study emphasizes the need to address these impairments, it remains unclear whether prehabilitation effectively improves gait. Future research should explore the potential of interventions such as strengthening exercises, gait retraining, and wearable sensor feedback to enhance gait mechanics and improve mobility in this population before surgery.

## Figures and Tables

**Table 1:** The demographic characteristics of the participants included in this study are presented as means and standard deviations for normally distributed variables and medians with interquartile ranges for non-normally distributed variables.

	Knee Osteoarthritis (*n* = 21)	Controls (*n* = 39)	*p* Value
Age (years)	73.57 ± 6.44	71.59 ± 4.81	0.229
Weight (kg)	82.29 ± 16.92	77.96 ± 9.66	0.270
Height (cm)	165.52 ± 7.23	163.81 ± 7.45	0.429
BMI	30.01 ± 5.75	29.15 ± 3.97	0.541
Shoe size (EU)	39.76 ± 2.26	39.81 ± 3.98	0.332 ᵃ
Gender, Male/Female, N (%)	3 (14.3)/18 (85.7)	10 (25.6)/29 (74.4)	0.704

ᵃ *p*-value based on Mann–Whitney U test; shoe size reported as median (IQR) due to non-normal distribution.

**Table 2:** Gait parameters in patients with severe knee osteoarthritis prior to total knee arthroplasty and in the non-arthritic control group are presented as means and standard deviations (SDs) for normally distributed variables and as medians with interquartile ranges (IQRs) for non-normally distributed variables.

Parameter	Knee Osteoarthritis	Controls		
	Mean ± SD	Mean ± SD	*p* Value	Effect Size (Cohen’s d)
Left gait cycle duration (s)	1.36 ± 0.14	1.11 ± 0.06	0.000	2.17
Right gait cycle duration (s)	1.35 ± 0.14	1.11 ± 0.07	0.000	2.17
Cadence (steps/min)	91.12 ± 9.02	108.78 ± 6.64	0.000	−2.23
Speed (m/s)	0.87 ± 0.16	1.11 ± 0.14	0.000	−1.60
Left stride duration (s)	1.37 ± 0.16	1.11 ± 0.06	0.000	2.15
Right stride duration (s)	1.36 ± 0.16	1.11 ± 0.07	0.000	2.02
Left stance duration (%)	60.95 ± 5.16	60.80 ± 2.34	0.892	0.04
Right stance duration (%)	60.66 ± 5.07	60.15 ± 2.90	0.663	0.12
Left stride length (m)	1.18 ± 0.20	1.23 ± 0.15	0.274	−0.28
Right stride length (m)	1.18 ± 0.19	1.23 ± 0.15	0.277	−0.29
First left double support (%)	11.18 ± 2.02	10.26 ± 2.38	0.163	0.42
Left single support (%)	39.14 ± 4.88	39.65 ± 2.67	0.646	−0.13
Right single support (%)	38.79 ± 5.22	39.10 ± 1.91	0.773	−0.08
Right swing duration (%)	39.34 ± 5.07	39.85 ± 2.90	0.663	−0.12
Left propulsion index	3.78 ± 1.47	6.45 ± 1.30	0.000	−1.92
Right propulsion index	4.01 ± 1.58	6.45 ± 1.42	0.000	−1.62
Right walk quality index	91.47 ± 5.30	94.91 ± 2.65	0.005	−0.82
	**Median** ± **IQR**	**Median** ± **IQR**	***p* value**	**Effect Size (r)**
Left elaborated steps (s)	11.0 ± 6.0	8.0 ± 2.0	0.000	0.66
Right elaborated steps (s)	11.0 ± 5.0	8.0 ± 2.0	0.000	0.69
Symmetry index of gait cycle	92.7 ± 14.85	97.0 ± 2.4	0.000	0.71
% Left stride length (% height)	69.1 ± 18.25	75.9 ± 11.7	0.143	0.17
% Right stride length (% height)	69.5 ± 17.65	75.4 ± 10.9	0.132	0.18
Left swing duration (%)	39.3 ± 6.6	38.5 ± 3.2	0.893	0.01
First right double support (%)	10.2 ± 3.1	11.8 ± 4.3	0.417	0.08
Left walk quality index	93.5 ± 6.8	95.9 ± 4.3	0.036	0.21
Tilt—symmetry index of pelvic angles	50.5 ± 49.1	74.2 ± 27.4	0.014	0.28
Obliquity—symmetry index of pelvic angles	90.3 ± 9.8	98.7 ± 0.8	0.000	0.52
Rotation—symmetry index of pelvic angles	94.8 ± 4.1	98.1 ± 2.4	0.002	0.40

## Data Availability

The original contributions presented in this study are included in this article. Further inquiries should be directed to the corresponding author.
